# Psoriasis and cardiovascular risk: associated and protective factors^☆^

**DOI:** 10.1016/j.abd.2024.07.013

**Published:** 2025-03-12

**Authors:** Luciana Agoglia, Helena Peixoto, Ana Carolina Cardoso, Lívia Barbosa, Cecília S.X.L. Victer, Sueli Carneiro, Gil F. Salles, Cristiane A. Villela-Nogueira, Maria Chiara Chindamo

**Affiliations:** a Department of Internal Medicine, Faculty of Medicine, Hospital Universitário Clementino Fraga Filho, Universidade Federal do Rio de Janeiro, Rio de Janeiro, RJ Brazil; b Section of Gastroenterology, Hospital Universitário Antônio Pedro, Universidade Federal Fluminense, Niterói, RJ, Brazil; c Dermatology Division, Hospital Federal de Bonsucesso, Rio de Janeiro, RJ, Brazil; d Dermatology Unit, Faculty of Medicine, Hospital Universitário Clementino Fraga Filho, Universidade Federal do Rio de Janeiro, Rio de Janeiro, RJ, Brazil

**Keywords:** Atherosclerosis, Fatty liver, Methotrexate, Psoriasis, Vascular stiffness

## Abstract

**Background:**

Psoriasis is an inflammatory skin disease associated with Metabolic Syndrome (MetS), Steatotic Liver Disease (SLD) and cardiovascular risk. However, the effect of anti-inflammatory therapy on cardiovascular risk is uncertain.

**Objectives:**

To determine the relationship between anti-inflammatory therapy and subclinical atherosclerosis in individuals with psoriasis, using the gold standard carotid-femoral Pulse Wave Velocity (cf-PWV) measurement. Additionally, to evaluate the association between cf-PWV, steatosis and Advanced Fibrosis (AF) using Transient Elastography (TE) by Fibroscan®.

**Methods:**

Cross-sectional study including psoriasis patients submitted to cf-PWV and TE. Steatosis was defined as a controlled attenuation parameter ≥ 275 dB/m, AF as liver stiffness measurement ≥ 10 kPa, and increased Aortic Stiffness (AoS) as cf-PWV ≥ 10 m/s. Significant cumulative methotrexate dose was ≥ 1500 mg (MTX1500). Logistic regression analysis evaluated the independent variables associated with increased AoS.

**Results:**

Eighty patients were included (mean age 56.2 ± 11.5-years, 57.5% female, BMI 28.6 ± 5.3 kg/m^2^). Prevalences of MetS, diabetes mellitus, dyslipidemia, systemic arterial hypertension, steatosis and AF were 57.5%, 40.0%, 67.5%, 70.0%, 50.0% and 16.3%, respectively. MTX1500 was present in 45%, immunobiological treatment in 33.8%, and cf-PWV ≥ 10 m/s in 21.2%. On logistic regression analysis, age was independently related to cf-PWV ≥ 10 m/s (OR = 1.21; 95% CI 1.06‒1.38; p = 0.003) and MTX1500 was a protective cardiovascular factor (OR = 0.18; 95% CI 0.038‒0.87; p = 0.033). No association was observed between steatosis, AF or immunobiological therapy and cf-PWV ≥10 m/s.

**Study limitations:**

Sample size.

**Conclusion:**

In patients with psoriasis, increased AoS was associated with age, but not with steatosis or AF. A protective cardiovascular effect of MTX was found in a psoriasis population with a high prevalence of MetS and its components.

## Introduction

Psoriasis is a chronic immune-mediated inflammatory skin disease, which prevalence in Western countries is around 2%‒4%,^1^ and is associated with Metabolic Syndrome (MetS).^2^ Psoriatic patients have a two-fold risk of Steatotic Liver Disease (SLD) compared to non-psoriatic controls,^3^ and it increases the risk of Advanced Fibrosis (AF), cirrhosis, and cardiovascular disease.^4^ Methotrexate (MTX) and Immunobiological (IB) therapy are effective anti-inflammatory strategies in psoriasis. On the last decades, liver fibrosis in psoriasis has been more associated with MetS and SLD than to the cumulative MTX dose.^5^, ^6^, ^7^

Long-term inflammatory status is associated with MetS and atherosclerosis.^8^ Aortic Stiffness (AoS), assessed primarily by carotid-femoral Pulse Wave Velocity (cf-PWV), is an accurate marker for subclinical atherosclerosis.^9^ Interestingly, it has been also described an association between increased arterial stiffness and advanced liver fibrosis, independent of other traditional cardiometabolic risk factors.^10^ In type 2 Diabetes Mellitus (T2DM) patients with SLD, a high or increasing aortic stiffness predicted the development of advanced liver fibrosis on Transient Elastography (TE).^11^

Thereby, considering the potential association between psoriasis, cardiovascular risk and SLD, the aim of this study was to evaluate the prevalence of subclinical atherosclerosis assessed by AoS and relationships with MTX and IB therapy. Advanced liver fibrosis and steatosis, assessed respectively by transient elastography (FibroScan®) and Controlled Attenuation Parameter (CAP), were additionally evaluated regarding the possible independent association with cardiovascular risk in psoriasis.

## Methods

### Study design and patients

This was a cross-sectional study from 2020 to 2022 of outpatients with established psoriasis diagnosis (clinically and/or histologically), followed by the dermatology division at two tertiary centers, Hospital Federal de Bonsucesso and Hospital Universitário Clementino Fraga Filho, with at least 18-years-old, regardless of the type of psoriasis specific treatment.

Exclusion criteria were: HIV, hepatitis B and hepatitis C infected patients, as well as those with other etiologies for chronic liver diseases, except Metabolic-Associated Steatotic Liver Disease (MASLD); use of hepatotoxic drugs in the last six months; use of steatogenic drugs (except MTX), like systemic corticosteroids, amiodarone, valproic acid and tamoxifen in the last two years or systemic chemotherapy in the last five years; daily alcohol intake greater than 20 g for women and 30 g for man in the last five years; conditions that could interfere with liver stiffness analysis (liver congestion, ascites, serum aminotransferase values greater than 5 times the upper normal limit, cholestasis and pregnancy). The Local Ethic Committee of both hospitals approved the study and all patients have signed an informed consent form.

### Study procedures

Individuals included in the study were submitted to anthropometric, clinical and laboratory evaluation, measurement of cf-PWV and liver stiffness /CAP measurements using TE. Blood sample collection for metabolic evaluation, Liver Stiffness Measurements (LSM), and the measurement of cf-PWV were performed on the same day.

### Demographic, clinical and laboratorial variables

Demographic (sex, age), anthropometric (Body Mass Index [BMI], abdominal circumference) and clinical (diagnosis of T2DM, systemic arterial hypertension, dyslipidemia, MetS according to ATPIII criteria)^12^ data were collected. Data regarding clinical psoriasis characteristics were time since psoriasis onset (time since the first cutaneous lesion onset, reminded by the patient), use of IB therapy (anytime) and cumulative MTX dose. Cumulative MTX doses ≥1500 mg were considered at risk for liver fibrosis.^5^ Laboratorial data included Alanine Aminotransferase (ALT), Aspartate Aminotransferase (AST), Gammaglutamyl Transferase (GGT), total cholesterol, HDL cholesterol, LDL cholesterol, triglycerides, glycated haemoglobin and platelet count. Liver enzymes (AST, ALT and GGT), were analyzed as absolute values and as indexes (absolute value/upper normal limit).

### Liver stiffness and controlled attenuation parameter measures

Liver stiffness measurement was performed with at least 3 hours of fasting by a single experienced operator using Fibroscan® TOUCH 502 (Echosens, France) with M and XL probes designed for this device. The used technique was previously described.^13^ Only results with 10 valid shots, success rate >60% and an Interquartile Interval (IQR)/median liver stiffness ratio <30% were included in the analysis. The results were expressed in kilopascals (kPa). CAP was simultaneously evaluated within valid LSM and was expressed in decibels per meter (dB/m). The XL probe, designed to evaluate measurements between 35 mm and 75 mm in depth (against 25‒65 mm in the M probe), was used in those patients who failed to obtain valid measurements with the M probe. A cut-off of 10 kPa was used to rule out advanced fibrosis^14^ and CAP results equal or greater than 275 dB/m defined the presence of steatosis.^15^

### Measurement of carotid-femoral pulse wave velocity (cf-PWV)

The cf-PWV was measured by the validated Complior SP device and software (Arthech Medical, Paris, France), by a single experienced operator. Carotid and femoral waveforms were recorded simultaneously using mechanotransducers applied directly to the skin, positioned in correspondence to the right carotid internal artery and right femoral artery. The software measured the difference in time (in milliseconds) elapsed between the beginning of the carotid and femoral pulse waves. The distance between the two points, the carotid-femoral distance (in centimeters), was measured directly and multiplied by 0.8.^16^ Two measurements were obtained from each patient, the result was the mean of the two measurements. If the difference between the two measurements was more than 0.5 m/s, a third measurement was taken. Cf-PVW was considered increased if ≥ 10 m/s.^16^

### Statistical analysis

Data was recorded in case report forms and entered in SPSS 21.0 software (IBM Corp, Armonk, New York). Categorical and continuous variables were analyzed and expressed as frequencies for categorical variables, means with standard deviations, and medians with interquartile intervals for continuous variables. Univariate analysis was performed using the Chi-Square or Fisher test for categorical variables, and Student's *t*-test or Mann-Whitney test for continuous variables, as appropriate. For the identification of variables independently associated with the presence of cf-PWV ≥ 10 m/s, binary logistic regression analysis was performed. The variables included in the model were those with clinical plausibility or p-values <0.20 on the univariate analysis. The level of significance adopted was 5%, with descriptive levels (p) below this value being considered statistically significant.

## Results

From 2020 to 2022, eighty patients were included (mean age 56.2 ± 11.5 years, 57.5% females, BMI 28.6 ± 5.3 kg/m^2^). The median duration of illness was 252-months (86‒383 months). All patients had successful cf-PWV and LSM measurements, as well as blood sample collection. Demographic, anthropometric, and clinical data are shown in Table 1. In univariate analysis, only age, T2DM, systemic arterial hypertension and cumulative MTX dose ≥ 1500 mg were associated with cf-PWV ≥10 m/s (the latter inversely associated) (Table 2). MetS, steatosis and advanced liver fibrosis were not associated with increased aortic stiffness. Variables included on logistic regression were age, sex, T2DM, systemic arterial hypertension, LSM (kPa) and cumulative MTX dose ≥ 1500 mg.

**Table 1:** Clinical-demographic and laboratorial data of patients with psoriasis.

Variables	n = 80
**Clinical-demographic characteristics**	
Female gender (%)	57.5
Age (years)	56.2 ± 11.5
BMI (Kg/m^2^)	28.6 ± 5.3
Abdominal circumference (cm)	101.7 ± 12.6
Type 2 Diabetes (%)	40.0
Arterial Hypertension (%)	70.0
Dyslipidemia (%)	67.5
Metabolic Syndrome (%)	57.5
Time since psoriasis onset (months)	252.0 (85.8‒383.3)
Cumulative MTX dose ≥ 1500 mg (%)	45.0
Immunobiological treatment (%)	33.8
**Laboratory**	
ALT index	0.47 (0.34‒0.67)
AST index	0.51 (0.44‒0.74)
GGT index	0.54 (0.37‒0.79)
LDL (mg/dL)	121.0 ± 37.0
HDL (mg/dL)	46.0 (37.5‒55.0)
Triglycerides (mg/dL)	133.5 (85.0‒182.5)
Platelet count (×10^3^)	256.5 ± 76.1
**Liver Transient Elastography**	
LSM (kPa)	5.6 (4.4‒8.7)
LSM (kPa) ≥ 10 (%)	16.3
CAP (dBm)	273.5 ± 51.9
CAP (dB/m) ≥ 275 (%)	50.0
**Carotid-femoral pulse wave velocity**	
cf-PWV (m/s)	8.8 ± 2.0
cf-PWV ≥ 10 m/s (%)	21.2

Values are proportions for categorical data, mean (SD) for normally distributed data and medians (interquartile intervals) for non-parametric data. BMI, Body Mass Index; ALT index, Absolute ALT value/upper normal limit; AST index, Absolute AST value/upper normal limit; GGT index, Absolute GGT value/upper normal limit; LDL, Low-Density Lipoprotein; HDL, High-Density Lipoprotein; LSM, Liver Stiffness Measurement; CAP, Controlled Attenuation Parameter; cf-PWV, Carotid-femoral Pulse Wave Velocity.

**Table 2:** Comparative analysis between patients with psoriasis with and without increased aortic stiffness (cf-PWV ≥ 10 m/s).

	cf-PWV < 10 m/s (n = 63)	cf-PWV ≥ 10 m/s (n = 17)	p-value
Age (years)	53.7 ± 11.2	65.7 ± 6.7	<0.001
Female gender (%)	58.7	52.9	0.66
BMI (Kg/m^2^)	28.9 ± 5.5	27.6 ± 4.6	0.18
Type 2 Diabetes (%)	31.7	70.6	0.004
Arterial Hypertension (%)	63.5	94.1	0.014
Dyslipidemia (%)	63.5	82.4	0.14
Metabolic Syndrome (%)	54	70.6	0.21
CAP (dB/m)	274.7 ± 49.6	269.1 ± 61.1	0.34
CAP ≥ 275 dB/m (%)	50.8	47.1	0.78
LSM (kPa)	5.4 (3.6)	7.1 (3.7)	0.20
LSM ≥ 10 kPa (%)	17.5	11.8	0.57
Time since psoriasis onset (months)	252.0 (299.4)	240 (289.5)	0.98
Cumulative MTX dose ≥ 1500 mg (%)	50.8	23.5	0.04
Immunobiological treatment (%)	36.5	23.5	0.31

Values are proportions for categorical data, means (SD) for normally distributed data and medians (interquartile ranges) for non-parametric data. Univariate analysis was performed using the Chi-Square or Fisher test for categorical variables, and Student's *t-*test or Mann-Whitney test for continuous variables. BMI, Body Mass Index; ALT index, Absolute ALT value/upper normal limit; AST index, Absolute AST value/upper normal limit; GGT index, Absolute GGT value/upper normal limit; N/A, Not Applicable; CAP, Controlled Attenuation Parameter; cf-PWV, Carotid-femoral Pulse Wave Velocity.

Beyond MTX use, age was the only other independent factor associated with cf-PWV ≥ 10 m/s. Cumulative MTX dose ≥1500 mg, presented in 45% of the population, represented a cardiovascular protective factor, but not IB use (Table 3).

**Table 3:** Final regression model for independently associated variables with the presence of increased aortic stiffness (cf-PWV ≥ 10 m/s).

Covariates^a^	Odds Ratio	p	95% CI
Age	1.21	0.003	1.06‒1.38
Cumulative MTX dose ≥ 1500 mg	0.18	0.033	0.038‒0.87

CI, Confidence Interval.

a Adjusted for age and sex.

## Discussion

This study, conducted on psoriasis patients with a high prevalence of MetS and its components such as T2DM, dyslipidemia, and systemic arterial hypertension, demonstrated a protective cardiovascular effect of cumulative MTX dose ≥1500 mg on subclinical atherosclerosis, using cf-PWV measurement for AoS. Although a high prevalence of SLD was found in this population, increased AoS was not associated with steatosis or AF.

Individuals with psoriasis have well-established increased arterial stiffness when compared to controls,^9^, ^17^, ^18^ and in most of the studies, it is independent of the effect of traditional risk factors, like smoking status, systemic arterial hypertension, and BMI. It suggests that psoriasis itself confers increased cardiovascular risk, probably due to chronic inflammation. The medium cf-PWV measurement in our study was 8.8 ± 2.0 m/s, and it was similar to most of the studies using cf-PWV for comparative analysis.^9^, ^19^ All previous studies in psoriasis were case-control, and none of them used the established recommendations for the measurement of cf-PWV, defined by a standard cut-off value of 10 m/s for the prediction of cardiovascular events.^16^ Therefore, our study was the first to report the prevalence of increased AoS using a standard value of cf-PVW ≥10 m/s on a population of psoriasis patients.

Our prevalence of PWV ≥ 10 m/s was 21.2%, similar to the 25% prevalence found on 477 patients with type 2 diabetes from the study nested within the Rio de Janeiro Type 2 Diabetes Cohort Study,^20^ also a population with high cardiovascular risk. Of note, increased AoS was not independently associated with MetS and its components in our study, and this corroborates with the hypothesis that psoriasis itself could increase cardiovascular risk.

As MASLD is itself a risk factor for atherosclerosis,^10^, ^21^ evaluation of aortic stiffness may be useful to predict both cardiovascular and liver fibrosis risk in this population. Increased aortic stiffness could be the “hallmark” to link the multiple inflammatory and cytokine-mediated mechanisms from the Hepato-Dermal Axis hypothesis,^22^, ^23^ as it reflects the long-term effects of established and unknown risk factors^18^ for cardiovascular and hepatic complications. Our study is the first to evaluate the association between arterial stiffness with SLD in psoriasis. Unfortunately, we could not show this link between liver fibrosis and early atherosclerosis, probably due to a rather small sample size. Hence, studies with larger sample sizes are necessary to better clarify this potential relation.

The independent association between age and increased AoS reflects the pathophysiological processes caused by aging on arterial walls extracellular matrix composition.^18^, ^24^ Curiously, the duration of disease in our study had no association with increased AoS, although it has been previously reported in a case-control study,^25^ which confirmed this association even after adjustment for confounders (age, weight, height, heart rate and central mean pressure). We could hypothesize that, in our study, patients using MTX had a benefit in cardiovascular risk, minimizing the effect of disease duration.

The protective effect of MTX on subclinical atherosclerosis in psoriasis found in our study is scarce in the literature. Using carotid or brachial intima-media thickness and endothelial function measurements, results with MTX and IB are divergent.^21^, ^26^ In our study, we evaluated subclinical cardiovascular risk (not cardiovascular events) with the gold standard method and could demonstrate the anti-inflammatory effect of MTX on cardiovascular risk, despite the high prevalence of cardiovascular co-morbidities like arterial hypertension, dyslipidemia and MetS itself in more than 50% of patients. This data highlights the role of inflammation in atherosclerosis regardless of the metabolic phenotype.^27^, ^28^ We could not demonstrate the same protective effect with IB therapy, probably due to the smaller number of patients using IB and/or the fact that it is a more recent therapy for psoriasis.

When analyzing cardiovascular events, three meta-analyses with patients presenting systemic inflammation (mainly rheumatoid arthritis),^29^, ^30^, ^31^ MTX treatment was associated with reduced incidence of cardiovascular events. On Horreau et al.^29^ systematic review, exclusively in psoriasis patients, two large retrospective studies found a protective effect of MTX on major cardiovascular events incidence. In one of them, treatment with MTX and biological agents was also associated with reduced risk of death and cardiovascular disease events in patients with severe psoriasis in a subsequent real-world analysis.^32^

There are some limitations in our study. The cross-sectional design does not allow causality to be proven. Disease severity was not measured, and associations were not performed regarding this variable. Sample size could have compromised the potential associations between liver fibrosis and early atherosclerosis, as well as a potential protective effect of IB on cardiovascular risk. Nevertheless, most of the case-control studies that established significantly higher AoS in psoriasis patients involved samples ranging from 20 to 73 patients.^18^

## Conclusion

A protective cardiovascular effect of MTX on subclinical atherosclerosis was found in a psoriasis population with a high prevalence of MetS and its components.

## Financial support

This study was supported by grants 429965/2018-4 from the National Council for Research and Technology, Conselho Nacional de Desenvolvimento Científico e Tecnológico (CNPq), Brazil.

## Authors’ contributions

Luciana Agoglia: Critical literature review; data collection, analysis and interpretation; preparation and writing of the manuscript; statistical analysis; study conception and planning.

Helena Peixoto: Data collection, analysis and interpretation.

Ana Carolina Cardoso: Intellectual participation in propaedeutic and/or therapeutic management of studied cases; approval of the final version of the manuscript.

Lívia Barbosa: Intellectual participation in propaedeutic and/or therapeutic management of studied cases; aproval of the final version of the manuscript.

Cecília S.X.L. Victer: Intellectual participation in propaedeutic and/or therapeutic management of studied cases; approval of the final version of the manuscript.

Sueli Carneiro: Intellectual participation in propaedeutic and/or therapeutic management of studied cases; approval of the final version of the manuscript.

Gil F. Salles: Manuscript critical review; approval of the final version of the manuscript.

Cristiane A. Villela-Nogueira: effective participation in research orientation; manuscript critical review; preparation and writing of the manuscript; statistical analysis; study conception and planning.

Maria Chiara Chindamo: Effective participation in research orientation; manuscript critical review; preparation and writing of the manuscript; statistical analysis; study conception and planning.

## Conflicts of interest

None declared.
