# Supplementary material for: Psoriasis and cardiovascular risk: associated and protective factors
Source: An Bras Dermatol. 2025 Mar 12;100(3):456–61. doi: 10.1016/j.abd.2024.07.013 (PMC12234172; doi:10.1016/j.abd.2024.07.013)
Supplement: Supplementary file 1 [file mmc1.docx]

**List of abbreviations:**

Pso, Psoriasis; MetS, Metabolic Syndrome; SLD, Steatotic Liver Disease; AoS, Aortic Stiffness; AF, advanced liver fibrosis; cf-PWV, carotid-femoral pulse wave velocity; LSM, liver stiffness measurement; MTX1500, cumulative methotrexate dose ≥ 1500mg; T2DM, Type 2 Diabetes Mellitus; MTX, Methotrexate; IB, immunobiological; BMI, Body mass index; CAP, Controlled attenuation parameter; ALT, Alanine aminotransferase; AST, Aspartate aminotransferase; GGT, Gamma-glutamyl transferase; HDL, high-density lipoprotein; LDL, low-density lipoprotein; IQR, Interquartile interval; kPa, Kilopascals; dB/m, Decibels per meter; MASLD, Metabolic Dysfunction-Associated Steatotic Liver Disease.
